# Implementation Research: Reducing the Research-to-Practice Gap in Depression Treatment

**DOI:** 10.1155/2012/476027

**Published:** 2012-12-26

**Authors:** Amy M. Kilbourne, Mark Williams, Mark S. Bauer, Patricia Arean

**Affiliations:** ^1^VA Ann Arbor Center for Clinical Management Research and Department of Psychiatry, University of Michigan Medical School, Ann Arbor, MI 48109, USA; ^2^Integrated Behavioral Health, Mayo Clinic, Rochester, MN 55905, USA; ^3^Center for Organization, Leadership, and Management Research, VA Boston Healthcare System and Department of Psychiatry, Harvard Medical School, Boston, MA 12130, USA; ^4^Department of Psychiatry, University of California, San Francisco, CA 94143, USA

“*The implementation gap prevents our nation from reaping the benefit of billions of US tax dollars spent on research and, more important, prolongs the suffering of millions of Americans who live with mental disorders*.”—President's New Freedom Commission on Mental Health 2003.

Despite the development of effective treatments, depression is still ranked as one of the top ten causes of disability worldwide according to the World Health Organization. It often takes years if not decades to translate evidence-based depression treatments into nonacademic, community-based practices. This research-to-practice gap can lead to millions of dollars of funded research being wasted when the treatments themselves never reach the populations in need [1]. Without a clear strategy to foster implementation and acceptance at the community provider level, a “voltage drop” is experienced when an evidence-based depression treatment developed in tightly-controlled academic settings is then introduced into real-world settings [2]. Moreover, current clinical trials populations are still highly selective (<5% of the diagnosed population) despite the movement towards “community-based research.” There is a paucity of effective strategies or implementation approaches for getting effective treatments for depression and other mental disorders off the academic shelf and into the hands of those who need them. 

Subsequently, there is a need for further cross-disciplinary research on effective processes to facilitate the uptake of evidence-based treatments in real-world practices. Implementation science is an emerging field poised to address the organizational, provider, and patient-level barriers to the adoption of new treatments for depression as well as for other conditions. The National institute of Mental Health defines implementation as the “use of strategies to adopt and integrate evidence-based health interventions and change practice patterns within specific settings [3].”

This current issue explores novel approaches based on implementation science for enhancing the uptake of depression treatments. The articles address a range of concepts, both at a higher level on tools for and theories of effective implementation and at the practical level of lessons learned on the ground. This series of articles also focuses on implementing evidence-based programs to improve depression outcomes particularly for underserved populations including lower income patients, adolescents, and women with postpartum depression.

Four articles describe the application of implementation theory and measures to inform changes in mental health practice in general and for depression treatment in particular. J. K. Benzer et al. describe barriers and facilitators to coordinated mental health care in primary care settings. Key findings were used to refine an implementation theory for coordinated depression care included the importance of organizational factors such as resources, provider training and workflow designs, and an understanding and appreciation of mental health and primary care practice boundaries. Two additional articles describe complementary approaches for engaging providers in the implementation of new mental health treatments. E. Aisenberg et al. describe a process for building a community-academic partnership to implement telephone cognitive-behavioral therapy for rural Latinos. The article points to how such partnerships can facilitate the adaptation of evidence-based practices in community settings by garnering frontline participant feedback on which elements need to be changed to implement a more scalable and culturally appropriate program. Similarly, D. E. Goodrich et al., using the Enhanced Replicating Effective Programs implementation framework, describe the implementation of an outreach program for Veterans with mental disorders, and what lessons were learned in regards to activating and empowering providers to “lead from the middle” and work within their systems to facilitate patient access. In addition, the paper by A. J. Lewis et al. describes how qualitative data can be applied to develop fidelity and implementation measures to assess update of a family-focused depression intervention. 

Providers are key stakeholders in the implementation process, yet few studies have focused on the development of effective implementation strategies to facilitate provider training in and sustainability of using evidence-based depression treatments. Three of the papers in this special issue address the challenges in training clinicians in new mental health treatments, and whether training leads to long-term adoption and use of these practices, in particular, problem-solving therapy (PST). J. A. Cartreine et al. describe a web-based training program to further disseminate PST training to novice clinicians and whether long-term coaching by more experienced providers to further boost training effectiveness might be needed. In the paper by R. M. Crabb et al., clinicians undergoing training in PST were followed over to assess the long-term impact of the training on adoption and spread. S. W. Stirman et al. identified key reasons for variation in cognitive behavioral therapy training outcomes across community-based practices and the importance of garnering feedback from frontline providers prior to implementing training initiatives.

Additional studies in this issue involved the implementation of depression treatments, notably in real-world populations. J. A. Waxmonsky et al. found that implementing a depression chronic care management programs in a public sector health plan led to significantly reduced depression severity over a 12-month period with a modest increase in health care costs. R. R. Dopp et al. present results of a novel program for adolescents with depression, demonstrating an alternative way to address mood symptoms that in turn also address other major chronic conditions such as obesity. Finally, B. P. Yawn et al. present a systematic review of postpartum depression treatment programs in which the most effective components included formal psychiatric evaluation, colocation of mental health care, and tracking of long-term outcomes.

Ultimately, implementation science can inform the adoption and adaptation of effective depression treatments, primarily through a better understanding of the importance of building partnerships with end-users such as health care organizations and frontline providers, understanding barriers to adoption of effective treatments at these levels, and developing interventions that use organizational strategies such as collaborative care or systematic training programs to facilitate adoption. Moreover, there will be additional opportunities for researchers and practitioners to apply the lessons learned from this emerging field to develop more effective and practical depression treatments from the beginning, as well as the use of provider and health care organization-level strategies to ensure their adoption and sustainability in the real world. To this end, new skills will need to be acquired, including the measurement of factors facilitating or impeding treatment uptake, especially at the provider and system level using both qualitative and quantitative methods, application of nonrandomized controlled study designs that take into account variations in provider and organizational differences, as well as the inclusion of real-world patient populations that to date are not well-represented in depression clinical trials. Moreover, by placing greater emphasis on partnerships, frontline provider input, and practical treatment strategies, mental health implementation researchers will be poised to lead the development of the next generation of effective depression treatments.

*Amy M. Kilbourne* *Amy M. Kilbourne* *Mark Williams* *Mark Williams* *Mark S. Bauer* *Mark S. Bauer* *Patricia Arean* *Patricia Arean*
